# Thoracic Spinal Angiolipoma: A Retrospective Case Series Highlighting Heterogeneous Imaging Presentations

**DOI:** 10.7759/cureus.116462

**Published:** 2026-09-17

**Authors:** Venkata Rakesh Babu Parimi, Venkata Shashank Davuluri, Visvanathan Krishnaswamy, Suprajaa Desingu, Bhaskar N P.

**Affiliations:** 1 Department of Neurosurgery, Sri Ramachandra Institute of Higher Education and Research, Chennai, IND; 2 Department of Neurological Surgery, Sri Ramachandra Institute of Higher Education and Research, Chennai, IND

**Keywords:** case series, magnetic resonance imaging, microsurgical resection, spinal angiolipoma, spinal cord compression, thoracic epidural tumor, thoracic myelopathy

## Abstract

Spinal angiolipomas are uncommon benign epidural tumors composed of mature adipose tissue and abnormal vascular channels. Their magnetic resonance imaging (MRI) appearance depends on the relative proportions of these components, which can make preoperative diagnosis difficult. The objective of this series is to describe the clinical, radiological, surgical, and histopathological spectrum of thoracic spinal angiolipomas and to discuss the implications of their imaging heterogeneity for preoperative diagnosis. We describe three adults with progressive thoracic myelopathy caused by posterior epidural lesions: a 53-year-old man with an eight-level T1-T8 fat-signal lesion without postcontrast enhancement; a 57-year-old man with a heterogeneously enhancing T4-T6 lesion causing severe canal stenosis; and a 65-year-old woman with a T5-T6 lesion with T6 vertebral marrow signal change that initially suggested vertebral hemangioma with epidural extension. Each patient underwent posterior decompression and microsurgical excision. Histopathology demonstrated mature adipose tissue admixed with benign vascular channels in the first two patients and an angiolipomatous vascular-fatty lesion with osseocartilaginous tissue in the third. All patients improved neurologically after surgery without a documented new deficit; follow-up MRI in the second patient at four months showed no residual lesion. This series illustrates the radiologic heterogeneity of spinal angiolipomas and the uncommon occurrence of long-segment disease. Recognition of the fatty and vascular components can narrow the differential diagnosis, but histopathology remains decisive. Adequate decompression and maximal safe resection generally provide favorable early outcomes.

## Introduction

Spinal angiolipomas are benign tumors composed of mature adipose tissue and abnormal vascular structures. They arise most often in the posterior epidural compartment and can compress the spinal cord or nerve roots. A 2026 systematic review of 240 patients found that 72.9% of lesions were thoracic, whereas long-segment involvement and hemorrhagic presentation were each reported in only 6.3% of cases [1]. A separate contemporary analysis also confirmed that back pain, paraparesis, and leg numbness are common presenting features and that most lesions involve two to four vertebral levels [2]. Although a female preponderance has traditionally been described, recent datasets show a more balanced sex distribution [1,2].

The etiology of spinal angiolipoma is poorly understood. Proposed predisposing or growth-modifying factors include obesity and weight gain, hormonal influences such as pregnancy, and, less consistently, trauma; however, these associations remain insufficiently established to be considered definitive risk factors [3,4]. No causal relationship between metabolic comorbidities and angiolipoma can be inferred, and none is implied by the comorbidity profile of the present series.

Magnetic resonance imaging (MRI) appearances vary with the relative amounts of fat and vascular tissue. Fat-rich lesions tend to be hyperintense on T1-weighted sequences and suppress on fat-saturated images, whereas vascular-rich lesions may be T1 isointense or hypointense and enhance strongly after contrast administration [3,5]. The Si et al. classification separates intraspinal lesions without paraspinal lipomatosis (type IA) from those with paraspinal lipomatosis (type IB); type II lesions have both intraspinal and extraspinal components [3]. Histopathologic examination remains necessary because the imaging differential includes lipoma, epidural lipomatosis, vertebral hemangioma with epidural extension, and other extradural tumors.

Posterior decompression with maximal safe excision is the standard treatment for symptomatic lesions and usually produces substantial neurologic recovery [6]. This imaging heterogeneity, in particular, long segment lesions and lesions with vertebral marrow signal change, both underrepresented in the literature, constitutes the knowledge gap this report addresses. The aim of this study is therefore to describe the clinical, radiologic, surgical, and histopathological features of three consecutive thoracic spinal angiolipomas, including an uncommon eight-level lesion and a lesion with vertebral marrow signal change that mimicked a hemangioma, and to discuss their implications for preoperative diagnosis.

## Case presentation

We reviewed clinical, imaging, operative, and pathology records of three consecutive adults treated at a single tertiary neurosurgical center between October 2023 and April 2026. We identified cases from the departmental operative register and cross-checked them against the histopathology database; these were the only histologically confirmed epidural angiolipomas operated on during this period, and we excluded no cases. Muscle strength was reported on the zero-to-five Medical Research Council (MRC) scale [7], and ambulatory function was graded retrospectively on the Nurick scale [8] from the documented clinical status at presentation and at the last review. Follow-up was defined as the interval from surgery to the last documented clinical review and is reported per patient; clinical and imaging follow-up are reported separately. Written informed consent for use of deidentified clinical, imaging, and histopathologic material was obtained from each patient or next of kin.

Case 1: T1-T8 long-segment angiolipoma

Clinical Presentation

A 53-year-old man with type 2 diabetes mellitus presented with a three-year history of progressive bilateral lower limb weakness that had worsened over one week, leaving him unable to walk independently. He had no trauma, fever, weight loss, known malignancy, or anticoagulant use. Sphincter function was preserved.

Examination

Both lower limbs had MRC grade 2/5 power, increased tone, brisk knee and ankle reflexes, and extensor plantar responses. Pain, temperature, and light-touch sensation were diminished below the lesion, whereas vibration and joint-position sense were preserved. The upper limbs were normal. Sphincter function was preserved. Nurick grade at presentation was 4.

Imaging

MRI demonstrated a fat-signal lesion in the left posterior epidural space from T1 to T8 without postcontrast enhancement. The lesion displaced the thoracic cord anterolaterally, with diffuse thinning over the corresponding levels (Figures 1, 2). On the contrast-enhanced sequence, the lesion retained high signal identical to that on the precontrast T1-weighted image, consistent with fat content rather than true enhancement (Figure 3). The documented preoperative working diagnosis was a compressive extradural extramedullary lesion; on imaging, the differential favored a fat-containing lesion, spinal lipoma vs. fat-predominant angiolipoma.

**Figure 1:**
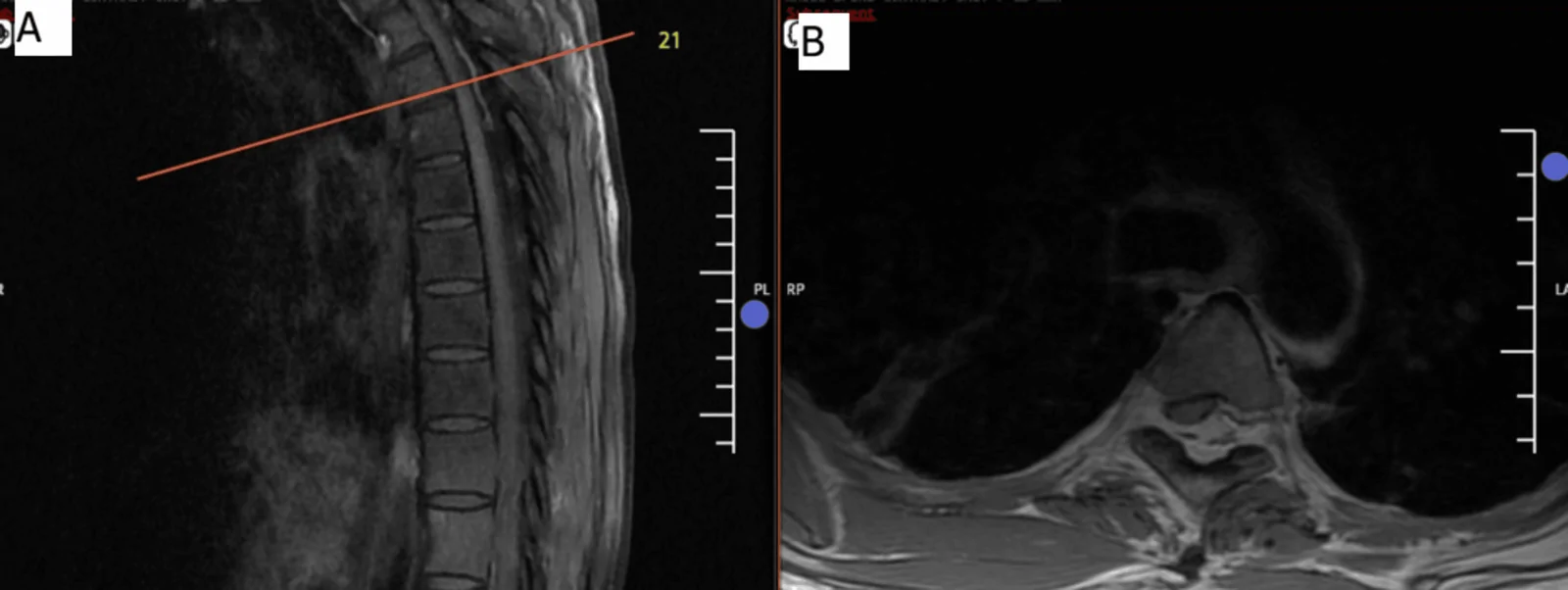
Thoracic MRI image of the first patient T1-weighted MRI sequence in (A) sagittal section showing a posteriorly placed epidural fat signal lesion extending from T1 to T8 vertebral level and (B) axial MRI demonstrating a posterior epidural lesion causing anterior displacement of the spinal cord MRI: magnetic resonance imaging

**Figure 2:**
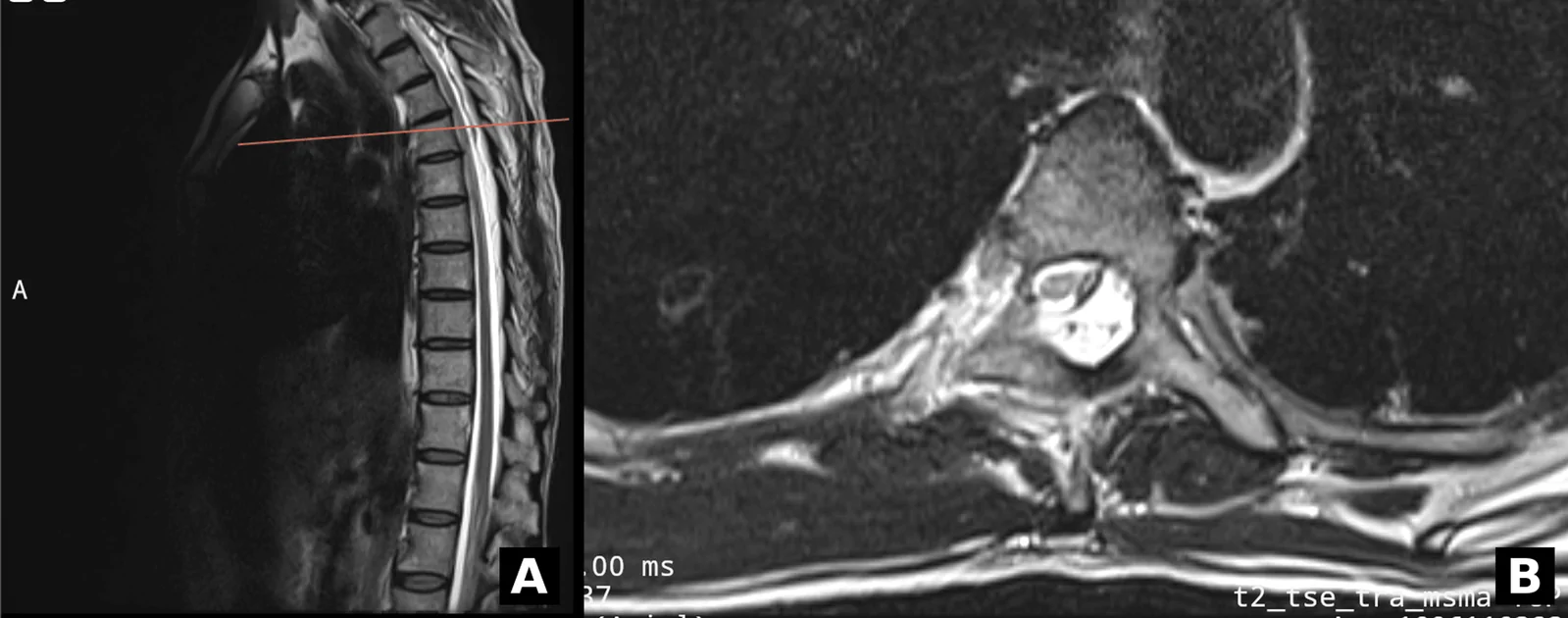
MRI image of the first patient T2-weighted MRI sequence in (A) midsagittal section showing a posterior epidural hyperintense lesion extending from the T1 to T8 vertebral levels, and (B) axial section at the T4 vertebral level showing an extra-axial lesion causing anterior displacement of the cord MRI: magnetic resonance imaging

**Figure 3:**
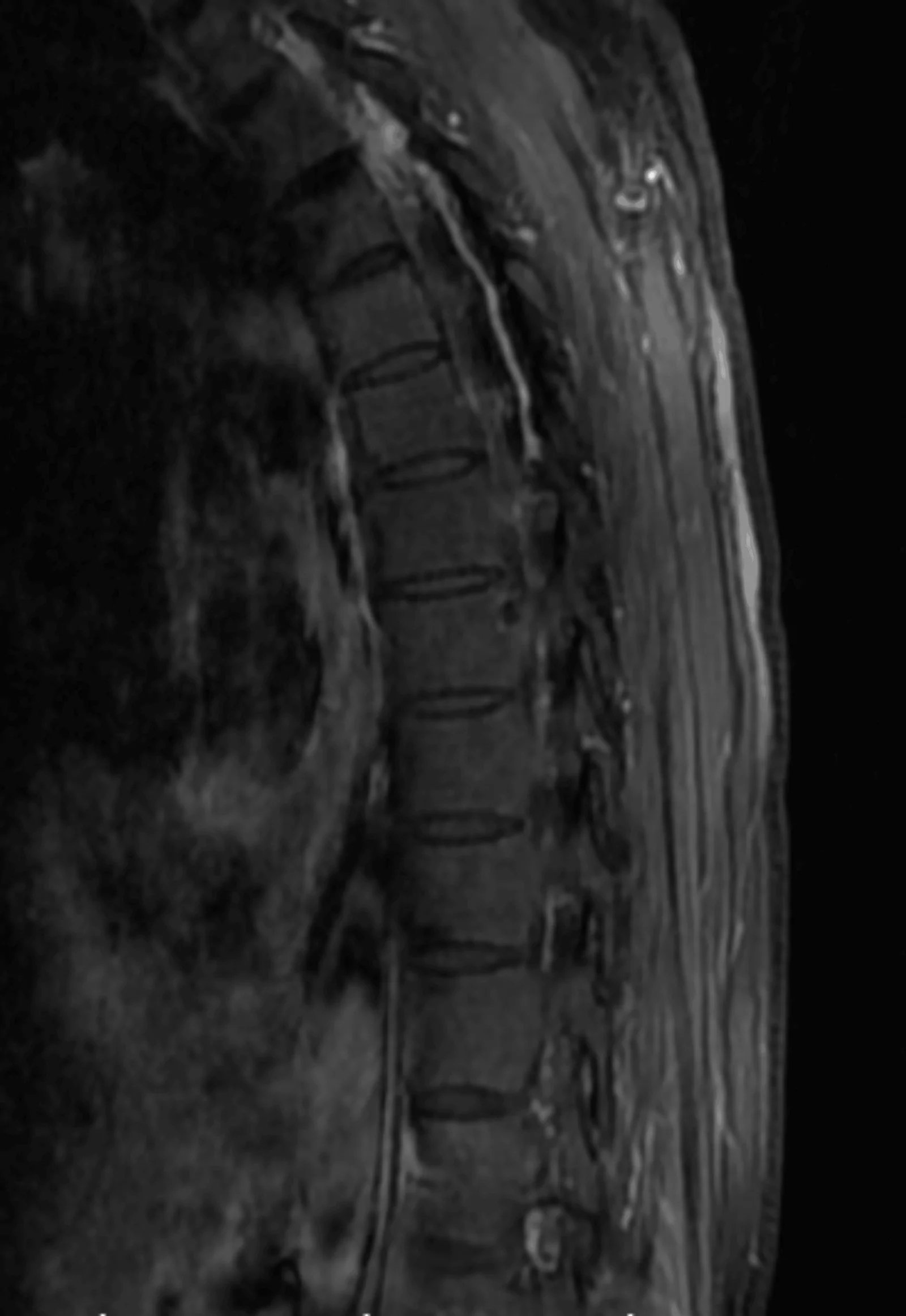
Thoracic MRI image of the first patient Contrast-enhanced T1-weighted MRI of the first patient, sagittal section. The posterior epidural lesion from T1 to T8 retains high signal identical to the precontrast T1-weighted image, indicating fat content rather than true postcontrast enhancement; the cord is displaced anteriorly MRI: magnetic resonance imaging

Surgery and Outcome

The patient underwent multilevel posterior thoracic laminotomy and microsurgical excision. The gray-red, soft extradural mass separated from the underlying dura, and gross total resection was achieved. The resected specimen comprised two gray-yellow to gray-brown soft-tissue fragments measuring 3.5 x 3 x 2 cm and 5 x 1 x 1 cm on gross examination. By postoperative day 1, lower limb power had improved to MRC grade 4/5 bilaterally. He mobilized with assistance from day 2 and walked independently at discharge. No perioperative complication was documented. At the last documented clinical review, 17 days after surgery, power was MRC grade 4/5 with independent ambulation (Nurick grade 0). No postoperative imaging was obtained during the documented follow-up period.

Histopathology

Hematoxylin and eosin-stained sections showed mature adipocytes admixed with proliferating capillary and cavernous vascular channels separated by hyalinized fibrous septa. Occasional bony spicules and small entrapped nerve bundles were present. No cytologic atypia, mitotic activity, or necrosis was identified, supporting a noninfiltrating spinal angiolipoma (Figure 4).

**Figure 4:**
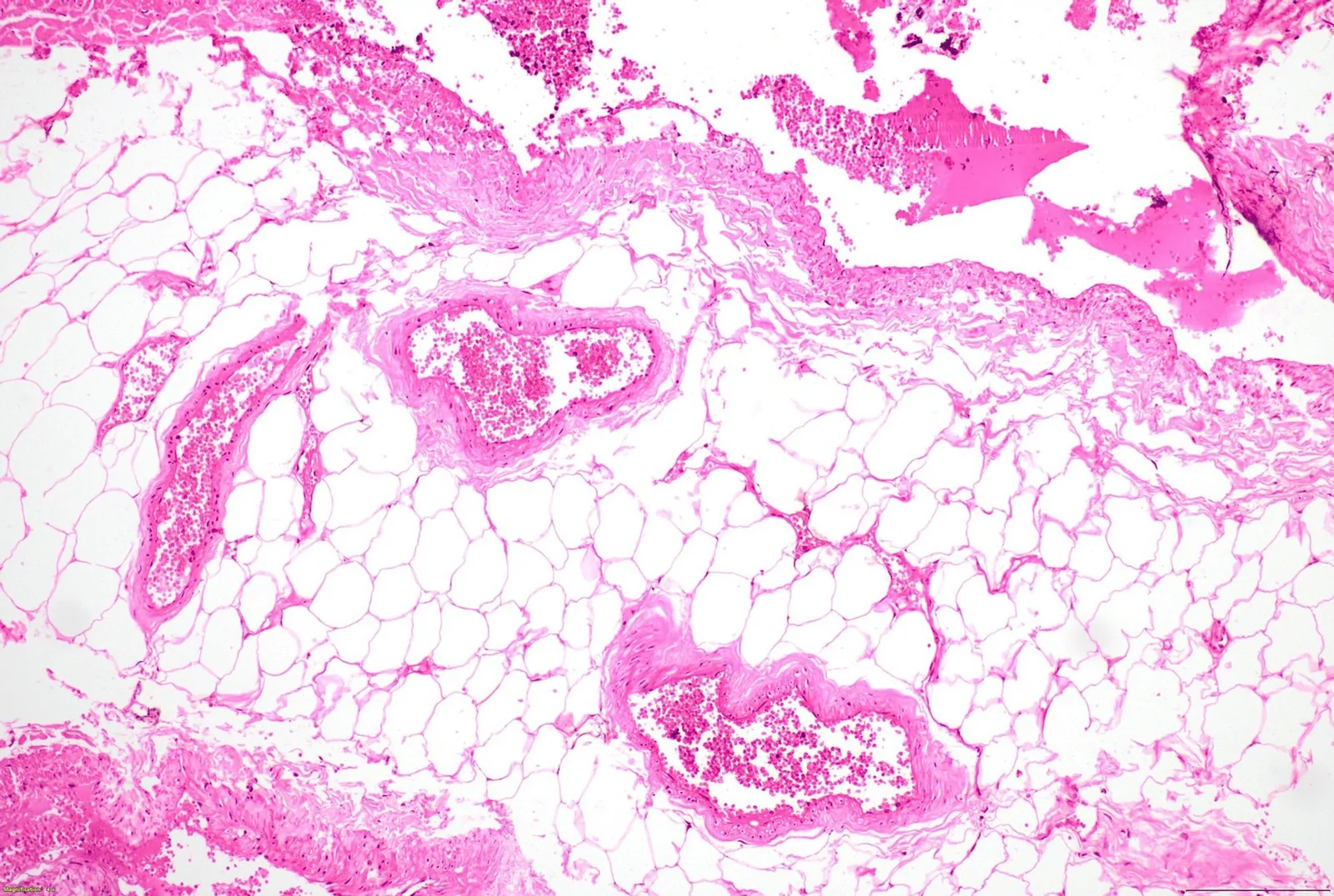
Histopathological slide derived from extra-axial spinal lesion of the first patient Hematoxylin and eosin-stained section at original magnification ×40 shows mature adipocytes and variably sized vascular channels separated by hyalinized fibrous septa

Case 2: T4-T6 angiolipoma with severe canal stenosis

Clinical Presentation

A 57-year-old man with hypertension and type 2 diabetes mellitus reported a 20-year history of low-back pain and progressive bilateral lower limb numbness, greater on the right. During the preceding several months, he developed difficulty rising from a chair and climbing stairs, a band-like lower abdominal sensation, knee buckling, unnoticed slippage of footwear, urinary urgency, and hesitancy. His body mass index was 33 kg/m^2^. There was no history of trauma or corticosteroid exposure.

Examination

He had bilateral lower limb paraparesis, greater on the right (MRC grade 4/5 on the right and 5/5 on the left), with increased tone, hyperreflexia, and extensor plantar responses. Pinprick and light-touch sensation were reduced at T5 and below, and vibration and joint-position sense were impaired distally. The upper limbs and cranial nerves were normal. Nurick grade at presentation was 2.

Imaging

Thoracic MRI demonstrated an ill-defined dorsal extradural lesion from T4 to T6 with mixed signal on T1- and T2-weighted sequences (Figures 5, 6) and corresponding hyperintensity on the short-tau inversion recovery (STIR) sequence (Figure 7). Heterogeneous postcontrast enhancement was present (Figure 8). Severe spinal cord compression and canal stenosis were greatest at T4-T5 and T5-T6 (Figure 6). The documented preoperative working diagnosis was a compressive extradural extramedullary lesion; on imaging, the heterogeneous enhancement favored a vascular epidural neoplasm.

**Figure 5:**
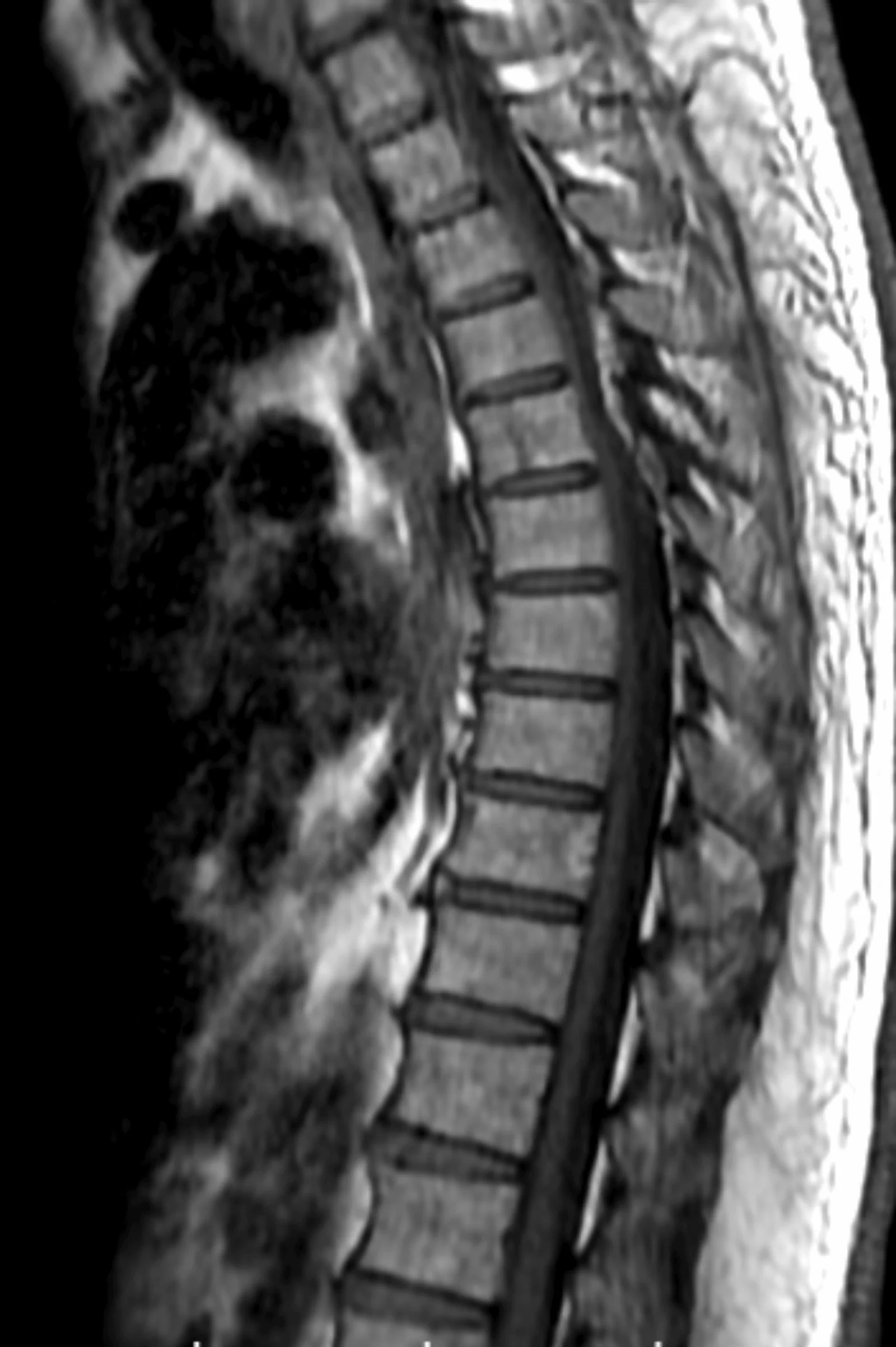
Thoracic magnetic resonance images of the second patient T1-weighted MRI sequences in sagittal section showing a dorsal extradural lesion from T4 to T6 with severe cord compression MRI: magnetic resonance imaging

**Figure 6:**
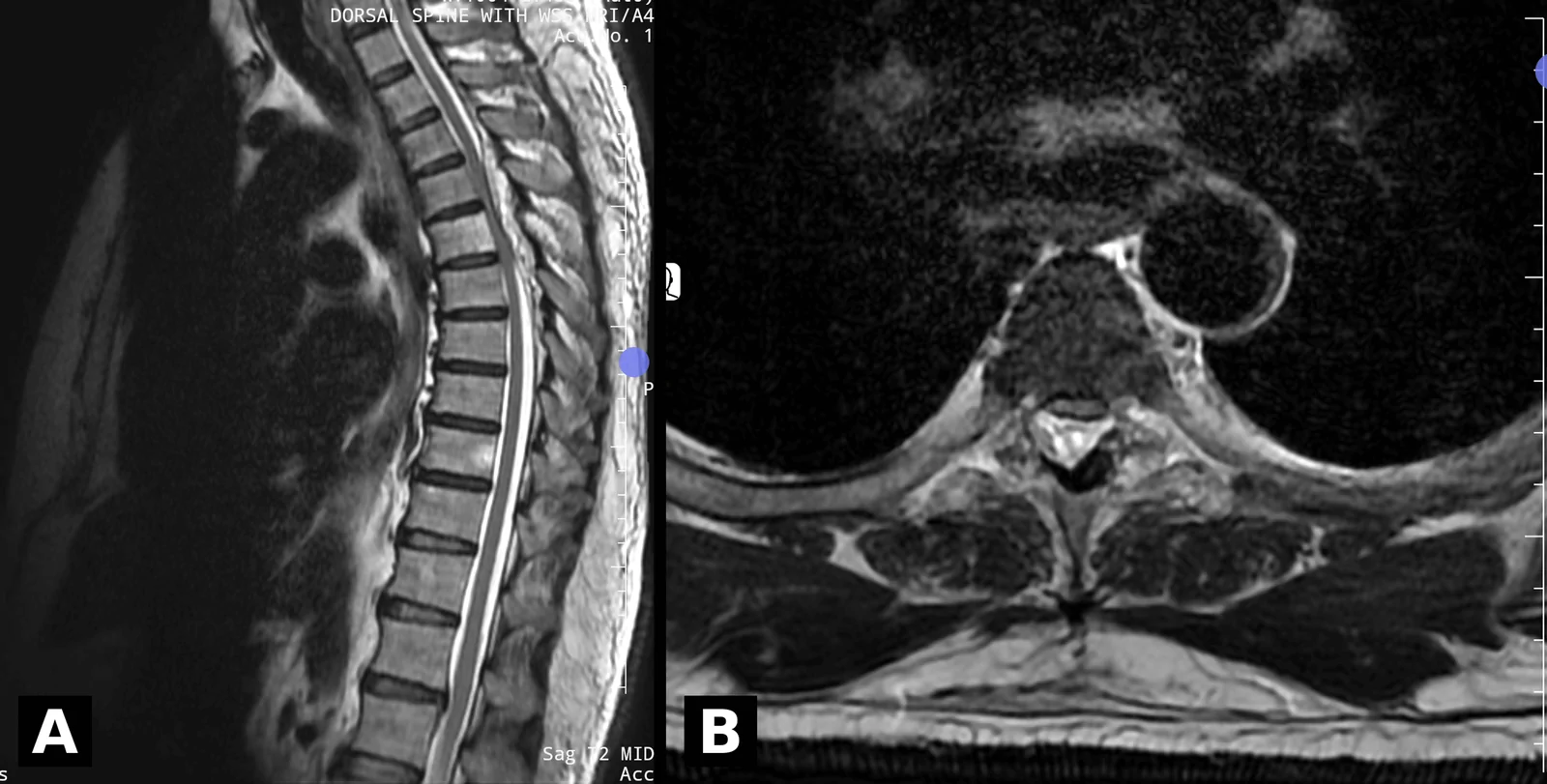
Thoracic magnetic resonance images of the second patient T2-weighted MRI sequences in (A) sagittal section and (B) axial section showing a dorsal extradural lesion from T4 to T6 with severe cord compression MRI: magnetic resonance imaging

**Figure 7:**
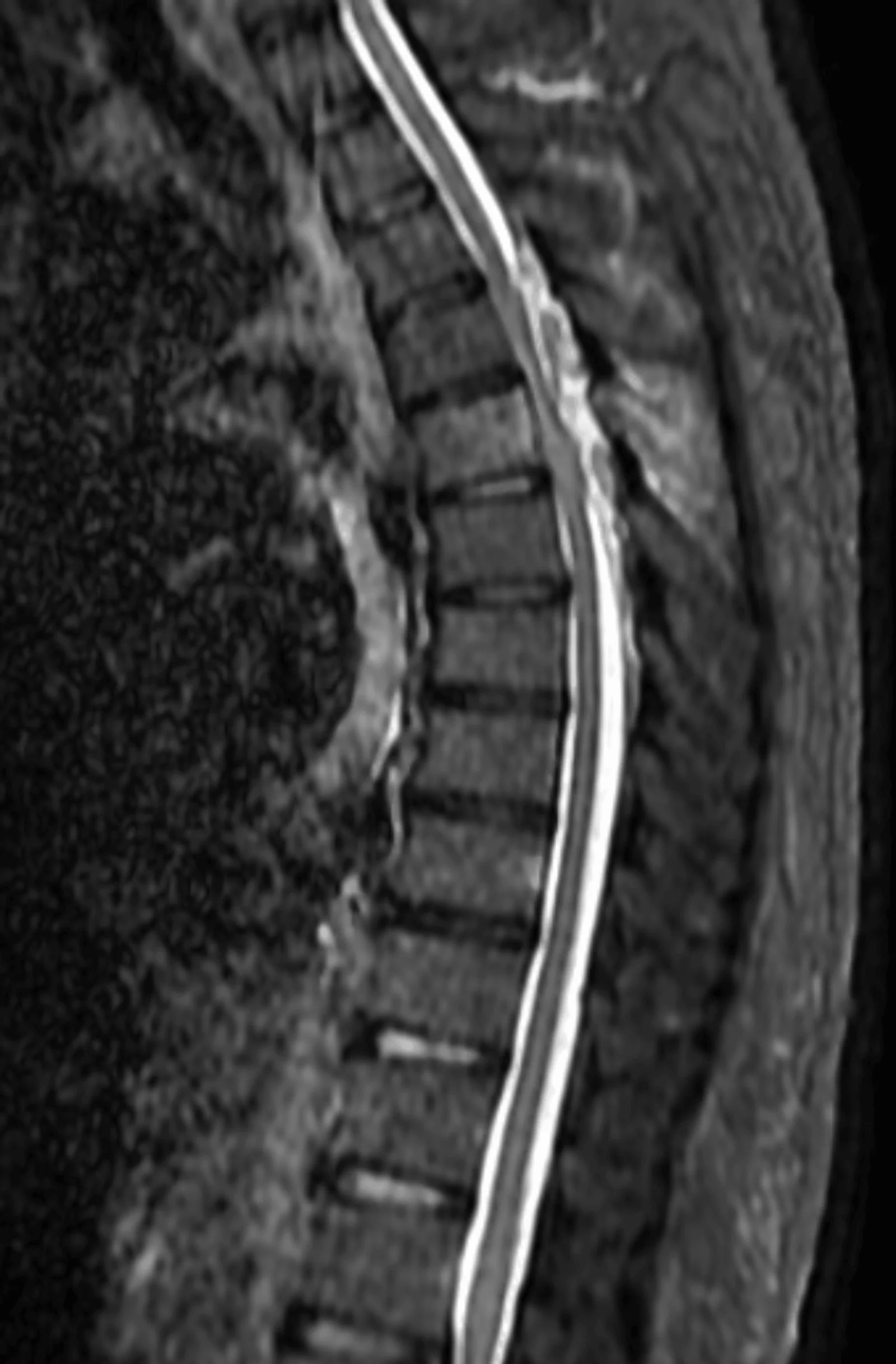
Thoracic magnetic resonance images of the second patient T2 STIR MRI sequence in sagittal section showing a dorsal extradural lesion from T4 to T6 with severe cord compression STIR: short-TI inversion recovery; MRI: magnetic resonance imaging

**Figure 8:**
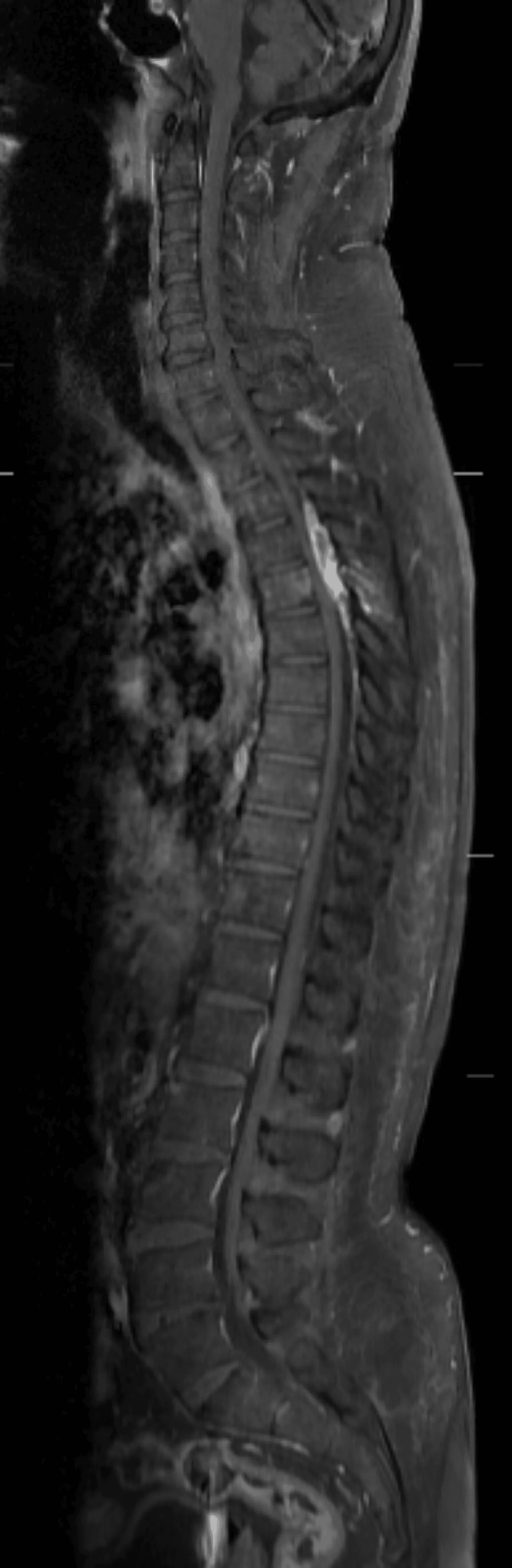
Whole spine magnetic resonance image of second patient Contrast-enhanced T1-weighted MRI sequence in sagittal section, showing a dorsal extradural lesion from T4 to T6 with severe cord compression and heterogeneous contrast enhancement MRI: magnetic resonance imaging

Surgery and Outcome

A T3-T6 laminectomy was performed, followed by microsurgical excision of the extradural lesion. The mass was gray-red, lobulated, and incompletely encapsulated but separated cleanly from the dura. Gross-total resection was achieved after bipolar control of recurrent radicular feeding vessels. The specimen comprised gray-white fragments with bony fragments measuring 2.5 x 2 x 1 cm and 3.5 x 2.8 x 1 cm and a gray-brown to gray-white soft-tissue fragment measuring 3 x 3 x 1 cm. Lower limb power improved to MRC grade 5/5 and the band-like sensation resolved. Follow-up MRI at four months showed no residual or recurrent lesion (Figure 9). At the last clinical review, almost at four months, he walked normally (Nurick grade 0).

**Figure 9:**
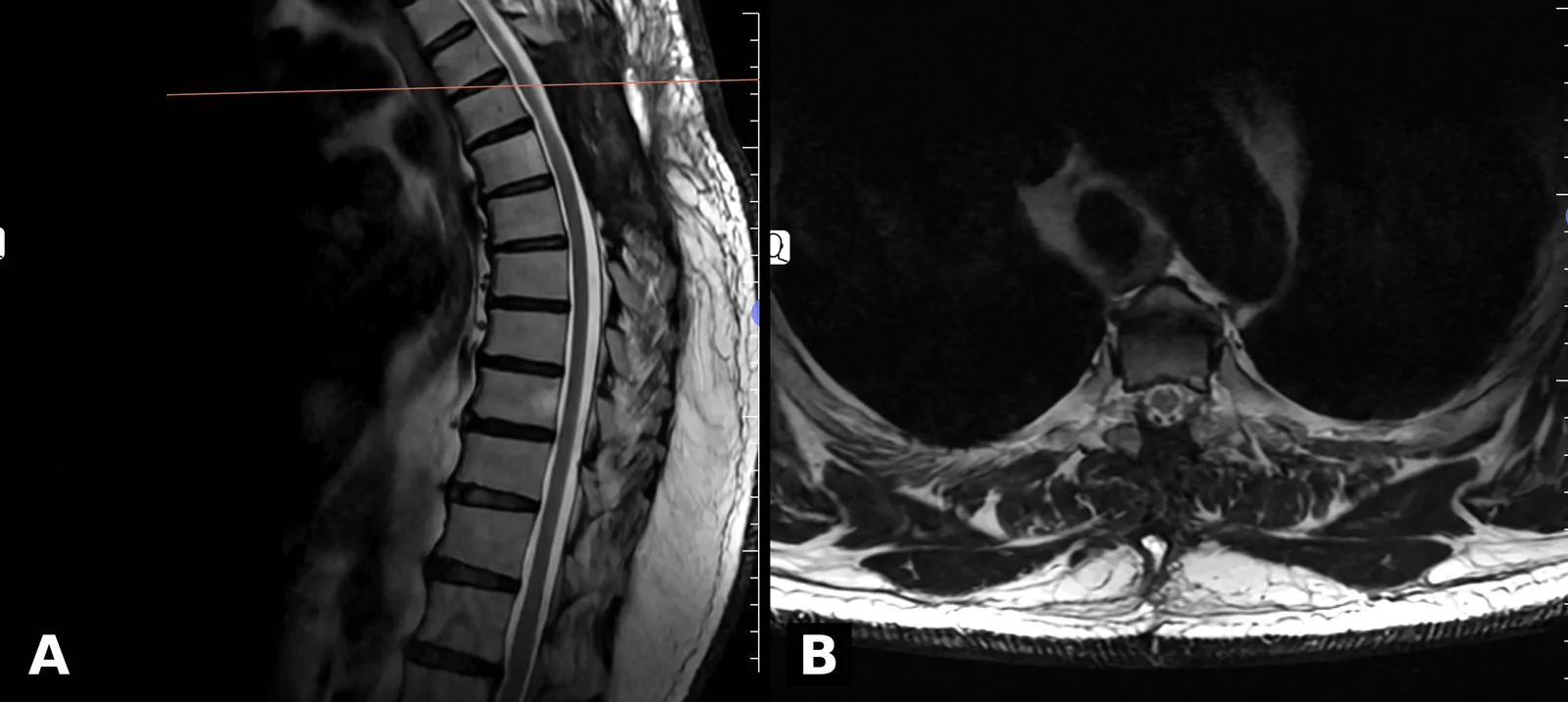
Postoperative MRI of the thoracic spine of the second patient Postoperative MRI imaging of the thoracic spine in (A) mid-sagittal section and (B) axial sections showing no residual lesion and postoperative soft tissue and bony changes MRI: magnetic resonance imaging

Histopathology

The specimen contained mature adipocytes and numerous small- to medium-sized thin-walled blood vessels containing erythrocytes, without atypia or mitotic figures. These findings confirmed a noninfiltrating spinal angiolipoma (Figure 10).

**Figure 10:**
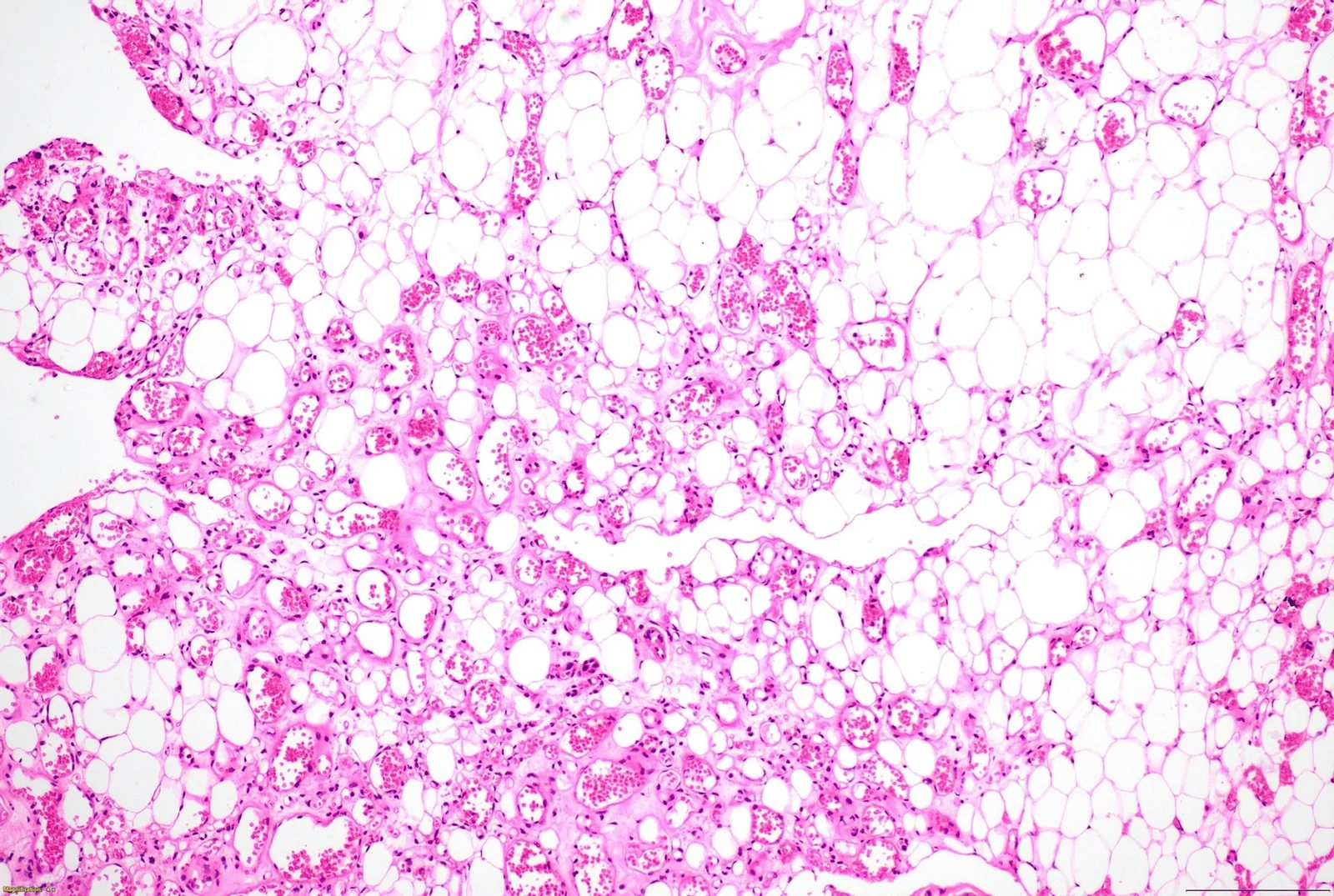
Histopathological examination findings in the second patient Hematoxylin and eosin-stained section at original magnification of ×40 showing mature adipocytes interspersed with numerous thin-walled vascular channels containing erythrocytes

Case 3: T5-T6 angiolipomatous lesion with vertebral marrow change

Clinical Presentation

A 65-year-old woman with hypertension, type 2 diabetes mellitus, and coronary artery disease presented with a six-month history of low-back pain radiating to both lower limbs, knee buckling, and difficulty rising from a chair. She had no bowel or bladder dysfunction, no history of trauma, and no hormonal or corticosteroid therapy.

Examination

She had bilateral lower limb paraparesis (MRC grade 4/5 bilaterally) with brisk reflexes and extensor plantar responses. Vibration and joint-position sense were impaired at the knees, ankles, and great toes, whereas pinprick and temperature sensation were relatively preserved. Sphincter function was normal. Nurick grade at presentation was 4.

Imaging

MRI showed a T1-hypointense and T2-hyperintense lesion at T5-T6 with avid postcontrast enhancement, associated marrow hyperintensity in the T6 vertebral body, and right paravertebral extension (Figures 11-14). Vertebral hemangioma with epidural extension was the main preoperative differential diagnosis. 18F-fluorodeoxyglucose positron emission tomography-computed tomography (PET-CT), performed to further characterize the vertebral lesion, showed a well-defined non-avid lesion centered on the T6 vertebral body and extending craniocaudally from T5 to T7, favoring a benign process; no bone biopsy or spinal angiography was undertaken.

**Figure 11:**
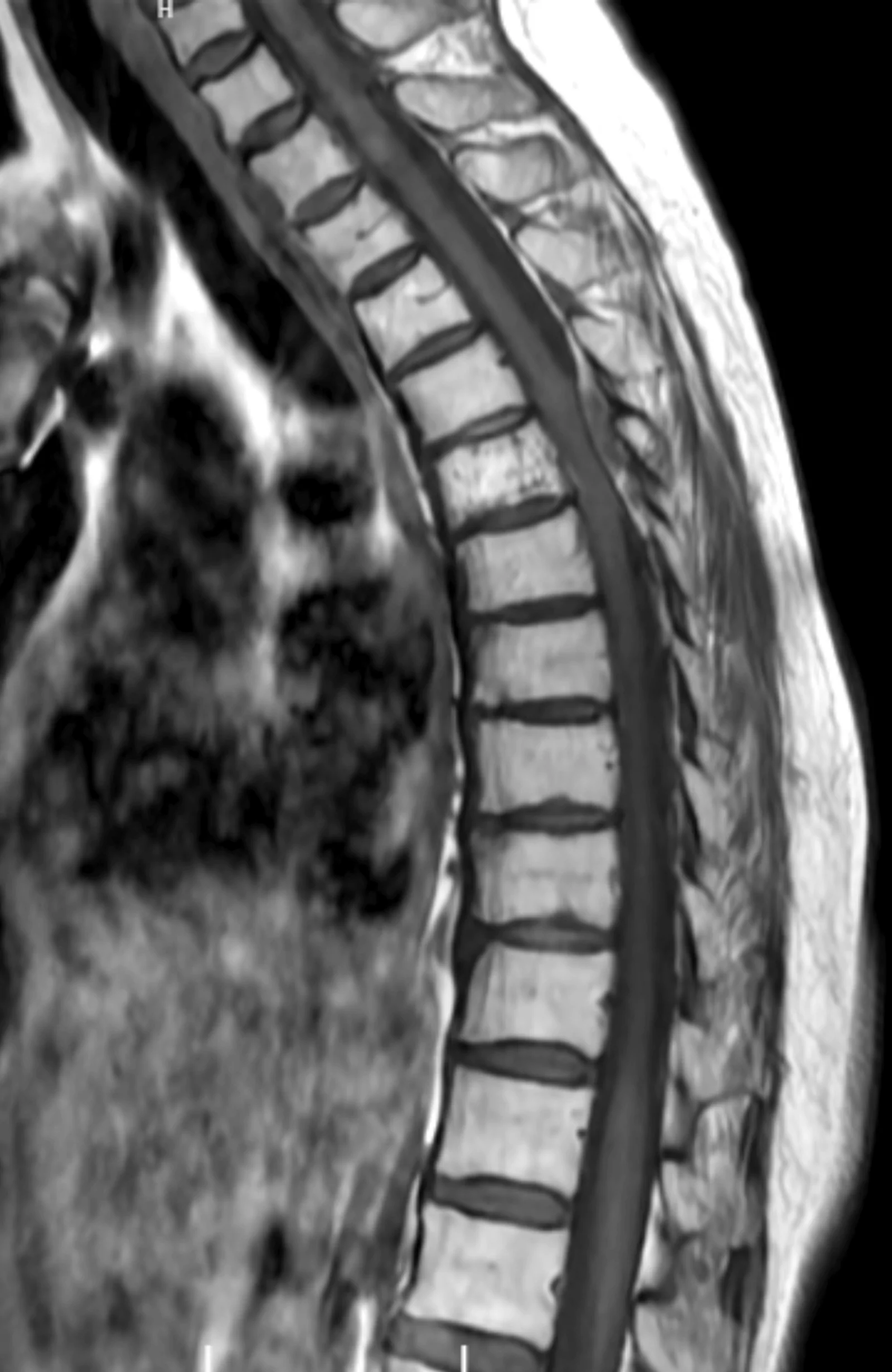
Thoracic magnetic resonance images of the third patient T1-weighted MRI sequences in sagittal section showing an extra-axial lesion at the T5-T6 vertebral level with T6 vertebral marrow signal changes compressing the thoracic spinal cord, with right paravertebral extension of the lesion MRI: magnetic resonance imaging

**Figure 12:**
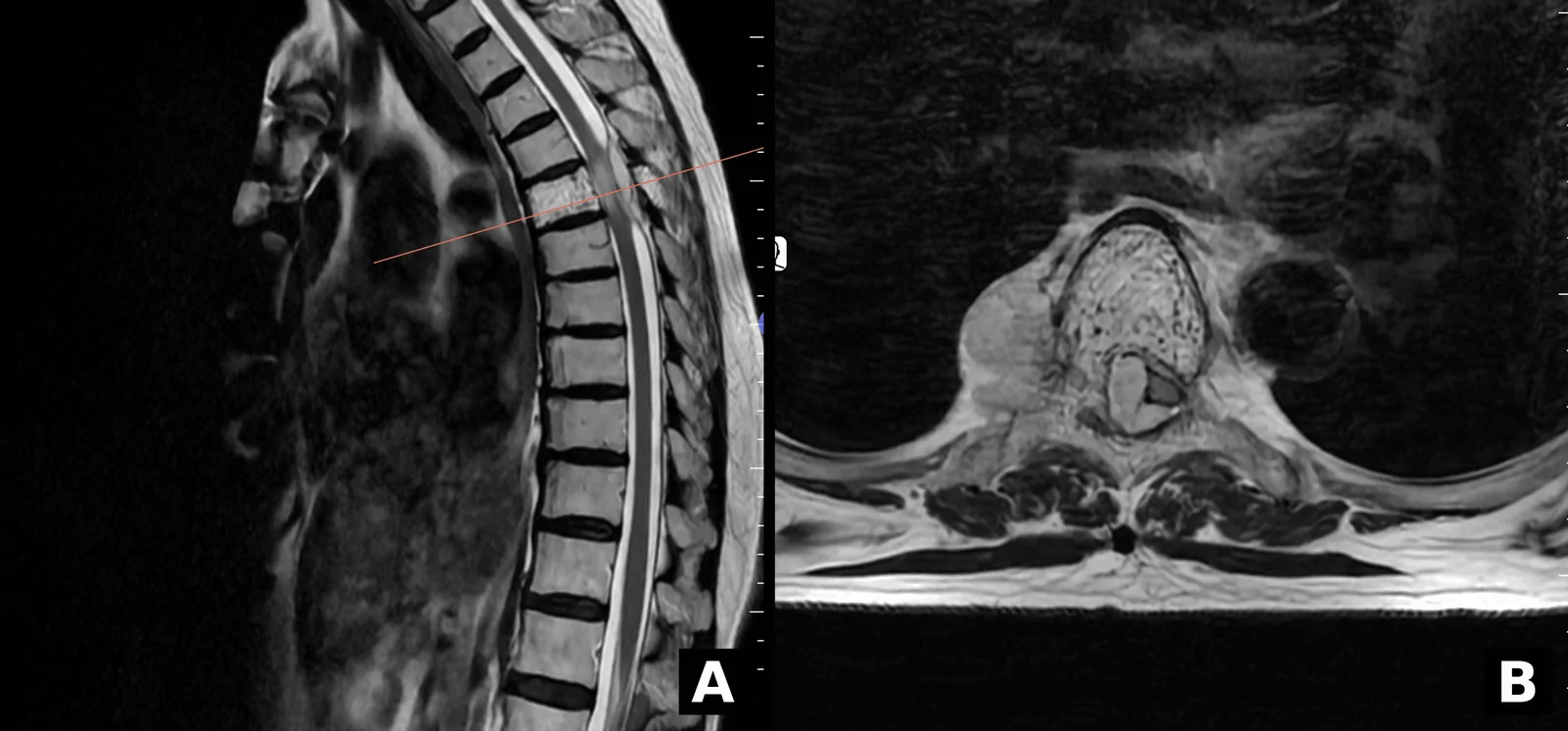
Thoracic magnetic resonance images of the third patient T2-weighted MRI sequences in (A) sagittal and (B) axial sections showing an extra-axial lesion from the T5-T6 vertebral level with T6 vertebral marrow signal changes compressing the thoracic spinal cord with right paravertebral extension of the lesion MRI: magnetic resonance imaging

**Figure 13:**
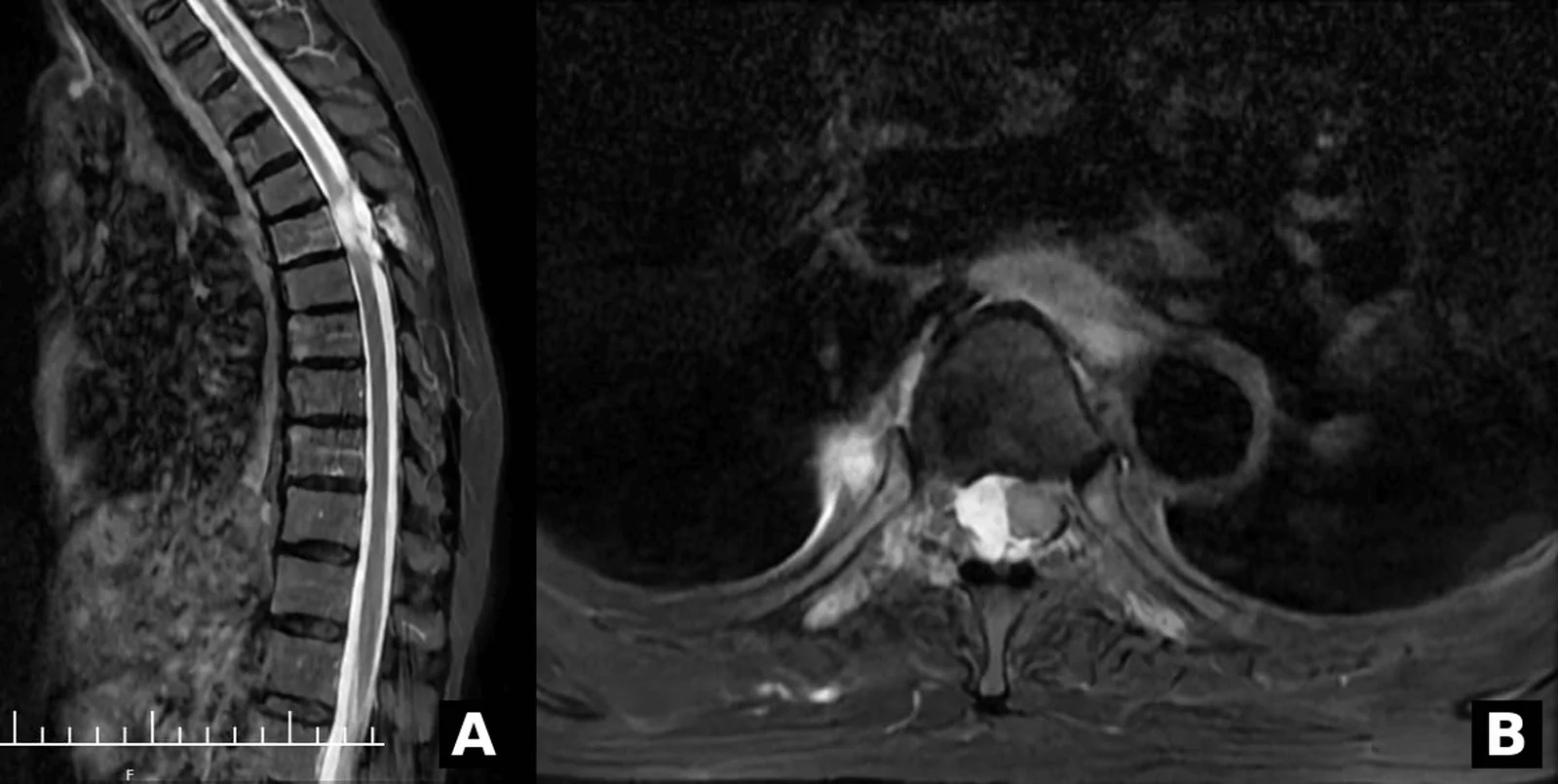
Thoracic MRI imaging of the third patient STIR MRI sequences in (A) sagittal and (B) axial sections showing an extra-axial lesion from the T5-T6 vertebral level with T6 vertebral marrow signal changes with right paravertebral extension and compression of the thoracic spinal cord STIR: short-TI inversion recovery; MRI: magnetic resonance imaging

**Figure 14:**
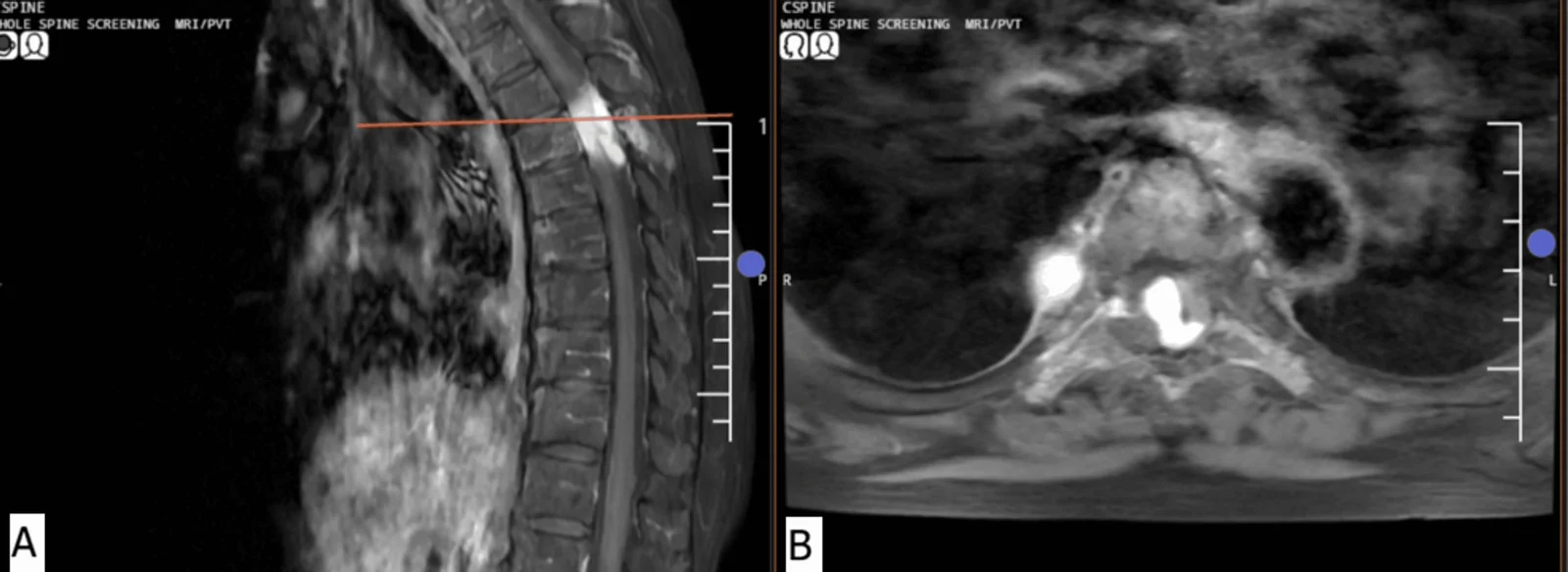
Thoracic magnetic resonance image of third patient Contrast-enhanced T1-weighted MRI sequences of the third patient. (A) Sagittal section showing an extra-axial lesion at the T5-T6 vertebral level with T6 vertebral marrow signal change and right paravertebral extension, compressing the thoracic spinal cord. (B) Axial section at the same level showing the avidly enhancing epidural component compressing the cord, with enhancement extending into the T6 vertebral body and the right paravertebral region MRI: magnetic resonance imaging

Surgery and Outcome

A T5-T6 laminectomy and microsurgical excision were performed. The lesion was vascular, soft, and well demarcated from surrounding tissue, and complete macroscopic excision was achieved; the vertebral body was not resected. The specimen comprised excised fragments of lesion from the T6 spinous process together with gray-white and gray-brown soft-tissue fragments measuring 1.5 x 1.8 x 1.8 cm and 2 x 1.3 x 0.8 cm. Lower limb power improved to MRC grade 5/5 with improved proprioception in the early postoperative period. At the last documented clinical review, eight days after surgery, she was ambulant (Nurick grade 0) with normal bowel and bladder function. No postoperative imaging was obtained during the documented follow-up period.

Histopathology

Sections showed variably sized vascular channels lined by bland endothelial cells within fibrous stroma, together with mature adipose tissue and osseocartilaginous fragments. Correlation with the imaging and operative findings favored a noninfiltrating angiolipomatous lesion adjacent to vertebral hemangiomatous change (Figures 15, 16).

**Figure 15:**
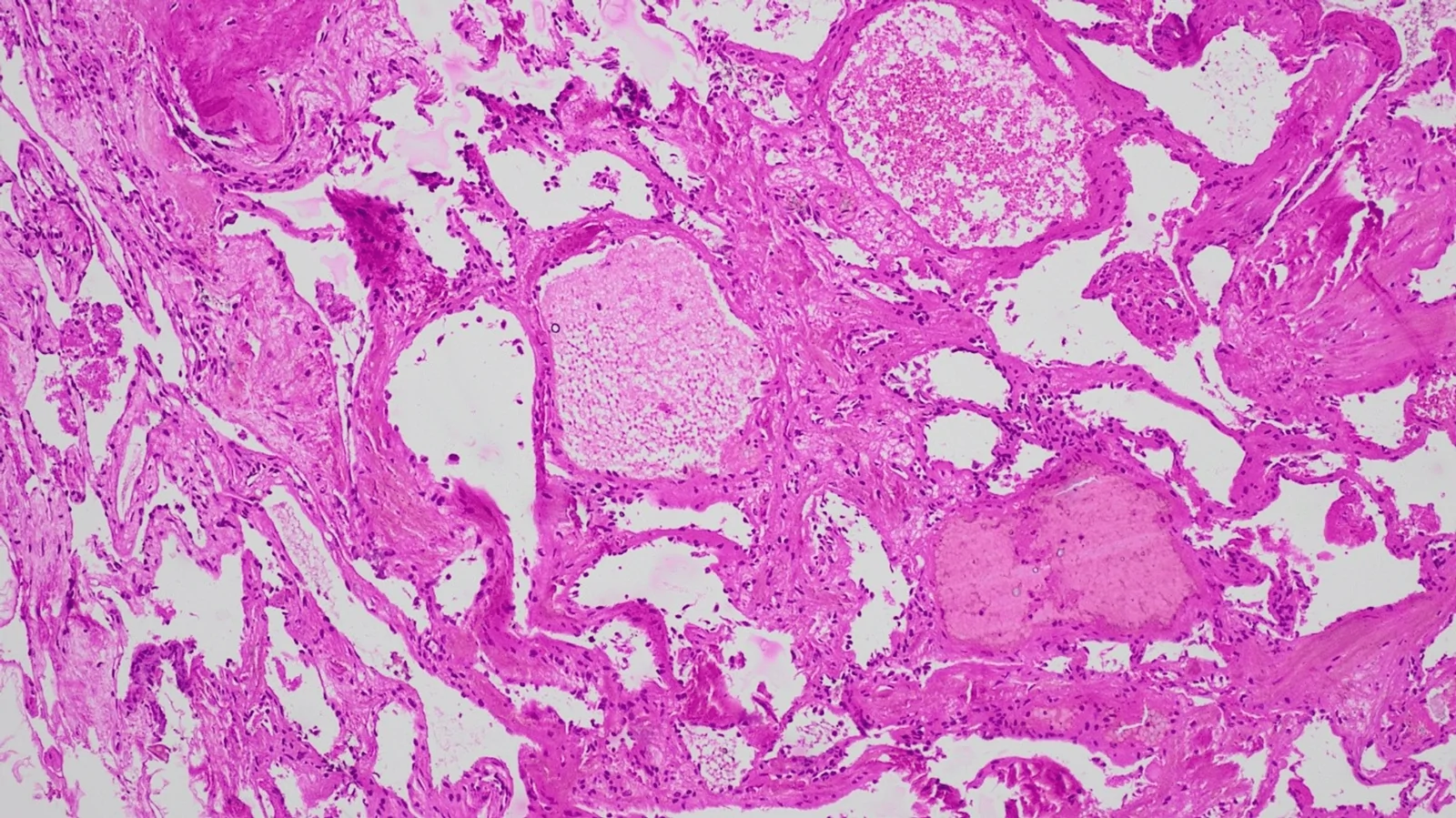
Histopathological findings in the third patient Hematoxylin and eosin-stained section at an original magnification ×10 showing variably sized vascular channels lined by bland endothelial cells within fibrous stroma, with adjacent osseocartilaginous tissue

**Figure 16:**
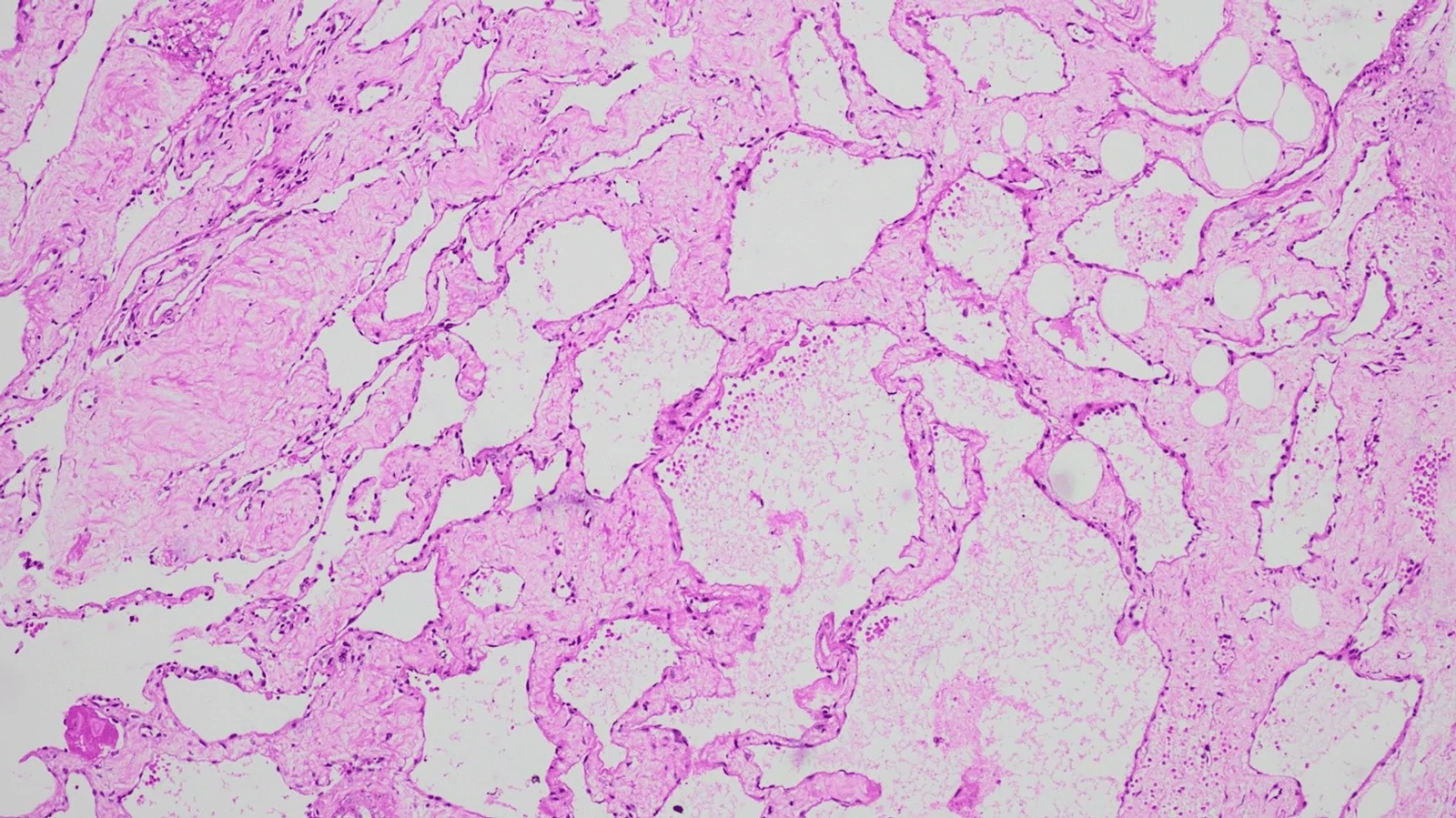
Histopathological findings of the third patient (angiolipomatous architecture) Hematoxylin and eosin-stained section showing mature adipose tissue admixed with interconnecting vascular channels lined by bland endothelial cells

The principal demographic, clinical, radiologic, operative, and histopathologic features of the three patients are summarized in Table 1.

**Table 1:** Clinical, radiologic, operative, and pathologic features of the three patients BMI: body mass index; CAD: coronary artery disease; DM: diabetes mellitus; FDG: fluorodeoxyglucose; MRC: Medical Research Council; MRI: magnetic resonance imaging; PET-CT: positron emission tomography computed tomography; T: thoracic

Variable	Patient 1	Patient 2	Patient 3
Age and sex	53-year-old man	57-year-old man	65-year-old woman
Comorbidities	Type 2 DM	Hypertension; type 2 DM	Hypertension; type 2 DM; CAD
BMI; steroid or hormonal exposure; trauma	BMI not documented; none; none	33 kg/m^2^; none; none	BMI not documented; none; none
Clinical course	Three years; acute worsening over one week	20 years; functional decline over several months	Six months; insidious low-back and bilateral leg symptoms
Key examination findings	Spastic paraparesis; MRC 2/5 bilaterally; impaired pain, temperature, and touch; sphincters preserved	Spastic paraparesis; MRC 4/5 right, 5/5 left; T5 sensory level; distal dorsal-column impairment; urinary urgency	Paraparesis; MRC 4/5 bilaterally; impaired vibration and proprioception; sphincters preserved
Nurick grade (presentation/last review)	4/0	2/0	4/0
Documented preoperative working diagnosis	Compressive extradural extramedullary lesion (imaging favored lipoma vs. angiolipoma)	Compressive extradural extramedullary lesion (imaging favored vascular epidural neoplasm)	Vertebral hemangioma with epidural extension
Lesion and MRI	T1-T8 posterior epidural fat-signal lesion; no enhancement	T4-T6 dorsal extradural lesion; mixed signal and heterogeneous enhancement	T5-T6 enhancing lesion; T6 marrow signal change; non-FDG-avid on PET-CT
Si et al. classification	Type IA	Type IA	Type II (intraspinal and extraspinal components)
Procedure	Multilevel laminotomy and microsurgical excision	T3-T6 laminectomy and microsurgical excision	T5-T6 laminectomy and microsurgical excision
Specimen (gross)	Two fragments, 3.5 × 3 × 2 cm and 5 × 1 × 1 cm	Fragments 2.5 × 2 × 1 cm, 3.5 × 2.8 × 1 cm (with bone) and 3 × 3 × 1 cm	Fragments from T6 spinous process; soft tissue 1.5 × 1.8 × 1.8 cm and 2 × 1.3 × 0.8 cm
Histopathology	Mature fat, capillary and cavernous channels, fibrous septa	Mature fat with numerous thin-walled vessels	Vascular-fatty lesion with fibrous and osseocartilaginous tissue
Outcome	MRC 4/5 by postoperative day 1; independent ambulation at discharge	MRC 5/5; band-like sensation resolved	MRC 5/5; improved proprioception; normal sphincters
Clinical follow-up (interval; status)	17 days; Nurick 0, MRC 4/5	Four months; Nurick 0, MRC 5/5	Eight days; Nurick 0, MRC 5/5
Imaging follow-up	None	MRI at four months: no residual lesion	None

## Discussion

Epidemiology, clinical presentation, and risk factors

The thoracic distribution of all three lesions is consistent with the largest contemporary synthesis, in which nearly three-quarters of lesions were thoracic. That review found motor weakness in 55.4% and pain in 42.9% of 240 patients. Long-segment disease and hemorrhagic presentation were each reported in only 6.3% of patients, and recurrence was uncommon, although follow-up was heterogeneous [1]. These findings contextualize the thoracic myelopathy in all three patients and the unusual eight-level extent in patient one. A three-patient series cannot be statistically compared with a 240-patient systematic review; these percentages are cited only to contextualize the present observations.

Most lesions in another large descriptive analysis involved two to four vertebral levels, and larger clinical cohorts have reported variable sex distributions [2]. Therefore, the presence of two men and one woman in this small series should not be interpreted as evidence of male predominance.

With respect to proposed risk factors, obesity and hormonal influences such as pregnancy have been discussed in the literature, although the associations remain unproven [3]. In this series, no patient had documented corticosteroid exposure or antecedent trauma; body mass index was documented only for patient two (33 kg/m^2^, in the obese range). The presence of metabolic comorbidities, including diabetes mellitus in all three patients, reflects the background prevalence of these conditions in this age group and permits no causal inference.

Imaging, classification, and management

The Si et al. classification describes intraspinal and extraspinal distribution, separating intraspinal lesions without paraspinal lipomatosis (type IA) from those with paraspinal lipomatosis (type IB); type II lesions have both intraspinal and extraspinal components [3]. The absence of paraspinal lipomatosis or a foraminal extraspinal component supports type IA classification for patients 1 and 2 despite the long craniocaudal extent in patient 1. Patient 3 had both intraspinal and extraspinal components, corresponding to Si et al. type II, although the vertebral marrow change was not sampled and its nature therefore remains uncertain. The classification carries a practical implication: dorsal type IA lesions are generally amenable to gross-total resection through a posterior approach, whereas type II or infiltrating lesions may require subtotal resection, staged or combined approaches, and closer postoperative surveillance, so subtype assignment informs both surgical planning and follow-up intensity [3,6].

MRI variability was the central diagnostic feature of this series. Fat-dominant spinal angiolipomas usually show high T1-weighted signal and signal suppression on fat-saturated sequences, whereas greater vascular content produces T2 hyperintensity and stronger enhancement [5]. Patient 1 had a long, fat-signal lesion without enhancement; patient 2 had mixed signal and heterogeneous enhancement; and patient 3 had low T1-weighted signal, high T2-weighted signal, avid enhancement, and vertebral marrow change. This spectrum demonstrates why a single MRI pattern cannot exclude the diagnosis.

Posterior decompression with maximal safe excision is the standard treatment for symptomatic lesions and generally produces substantial neurologic recovery. Gross-total resection is usually achievable for dorsal, noninfiltrating lesions. When infiltration or ventral extension makes complete excision unsafe, adequate decompression with subtotal resection can still provide good neurologic recovery, and routine postoperative radiotherapy is not recommended for a benign residual lesion [6]. All three patients underwent posterior decompression and complete macroscopic excision, followed by neurologic improvement.

Differential diagnosis and histopathology

A fat-containing posterior epidural lesion raises consideration of spinal lipoma, epidural lipomatosis, and angiolipoma. Marked enhancement suggests a vascular component, while vertebral marrow involvement broadens the differential to vertebral hemangioma with epidural extension and infiltrating angiolipoma. Infiltrating lesions can contain less visible fat, appear T1 hypointense, and involve adjacent bone [9]. For patient 3, the well-demarcated operative plane favored noninfiltrating behavior, and the absent fluorodeoxyglucose avidity on PET-CT supported a benign process, although it could not distinguish hemangioma from angiolipoma. The synchronous epidural and vertebral findings in this patient are best explained either as an aggressive vertebral hemangioma with an epidural angiolipomatous component or as an angiolipomatous epidural lesion adjacent to incidental vertebral hemangiomatous change; because the vertebral body was not resected or biopsied, the marrow process could not be confirmed histologically, and the marrow signal and osseocartilaginous tissue therefore require radiologic-pathologic correlation.

The diagnostic histologic pattern is mature adipose tissue admixed with benign vascular channels of variable caliber [10]. Absent cytologic atypia, mitotic activity, and necrosis support benignity. Patients 1 and 2 had the classic pattern. Patient 3 had a more vascular and fibrous lesion with osseocartilaginous fragments, but mature fat and bland vascular channels, together with the intraoperative appearance, supported an angiolipomatous lesion.

Comparison with published literature

The features of this series concordant with published reports are the dorsal thoracic epidural location, presentation with progressive myelopathy and back pain, resectability through a posterior approach, and early neurologic improvement after decompression [1,2,6]. Two observations are uncommon relative to the literature. First, the eight-level lesion in patient 1 contrasts with the two-to-four-level extent typical of published cohorts [2] and with the 6.3% frequency of long-segment disease in the 2026 systematic review [1]; reported long-segment cases, including recurrent long-segment disease with mediastinal extension, remain rare [1]. Second, vertebral marrow signal change accompanying an epidural angiolipomatous lesion, as in patient 3, is an unusual presentation that overlaps radiologically with vertebral hemangioma with epidural extension and with infiltrating angiolipoma [9]. The early functional outcomes observed here are broadly similar to those in published surgical series [11].

Clinical course, outcomes, and surveillance

Patient 1 illustrates accelerated deterioration after a long indolent course. Sudden or subacute worsening can occur because of intratumoral hemorrhage or thrombosis [12]. Neither was documented radiologically or histologically in this patient; progressive cord compression with loss of physiologic reserve is therefore a more cautious interpretation.

Published series likewise report favorable functional outcomes after surgery [4,11-13]. Long-segment exposure requires attention to preservation of the facets and posterior tension band, particularly in patients with many levels of decompression. Because the literature indicates that recurrence is uncommon rather than impossible and long-term follow-up remains limited [1,6], clinical surveillance and interval MRI are reasonable recommendations drawn from the published literature rather than from this series, especially after subtotal resection, in infiltrating disease, or when the pathologic classification is uncertain, as in patient 3.

Limitations

This retrospective, single-center series contains only three patients and cannot estimate demographic patterns, recurrence risk, or comparative treatment effects. The cases are also subject to selection bias: they were highlighted precisely because of their heterogeneous imaging characteristics, so the series illustrates the diagnostic spectrum rather than the typical distribution of presentations. MRI protocols were not uniform, and scanner acquisition parameters were not uniformly retrievable from the archived studies; an STIR sequence was available for patients 2 and 3 but not for patient 1. Documented neurologic examinations were not uniform, and the Nurick grade was assigned retrospectively from the recorded ambulatory status rather than prospectively. Clinical follow-up was short and unequal, 17 days for patient 1 and eight days for patient 3, against four months for patient 2, and postoperative imaging was documented only for patient 2, so no conclusion about recurrence can be drawn from this series. Body mass index was documented only for patient 2. In patient 3, the vertebral process was characterized noninvasively (PET-CT) but not biopsied, so the marrow diagnosis rests on radiologic-pathologic correlation alone; together with the mixed osseocartilaginous elements, this limits confidence in determining whether the vertebral marrow change represents incidental hemangiomatous change or extension of the angiolipomatous lesion. These limitations should temper interpretation of the descriptive observations.

## Conclusions

The key clinical lesson of this descriptive series is that no single MRI pattern excludes spinal angiolipoma: a posterior epidural lesion that contains fat, enhances after contrast administration, or produces progressive thoracic myelopathy should keep the diagnosis in the differential, even when the appearance is atypical, such as a nonenhancing long-segment fat-signal lesion or an avidly enhancing lesion with vertebral marrow signal change mimicking hemangioma. Diagnosis should integrate MRI, operative findings, and histopathology, and the diagnostic uncertainty that can remain when bone is not sampled, as in our third patient, should be acknowledged. In these three patients, posterior decompression with maximal safe resection was followed by early neurologic improvement, consistent with published series, although follow-up in this series was short. Recommendations regarding surveillance derive from the published literature rather than from this small series: because recurrence is reported as uncommon rather than impossible, follow-up is reasonably tailored to completeness of resection, infiltrative behavior, and diagnostic certainty.
